# Analysis of the burden and economic impact of digestive diseases and investigation of research gaps and priorities in the field of digestive health in the European Region—White Book 2: Executive summary

**DOI:** 10.1002/ueg2.12298

**Published:** 2022-09-12

**Authors:** Tanith C. Rose, Andy Pennington, Chris Kypridemos, Tao Chen, Moeez Subhani, Johanna Hanefeld, Luigi Ricciardiello, Ben Barr

**Affiliations:** ^1^ Department of Public Health, Policy and Systems Institute of Population Health University of Liverpool Liverpool UK; ^2^ Department of Global Health and Development Faculty of Public Health and Policy London School of Hygiene and Tropical Medicine London UK; ^3^ Department of Medical and Surgical Sciences IRCCS Azienda Ospedaliero Universitaria di Bologna Bologna Italy

**Keywords:** digestive system diseases, digestive system neoplasms, economics, epidemiology, public health, research funding, socioeconomic factors

## Abstract

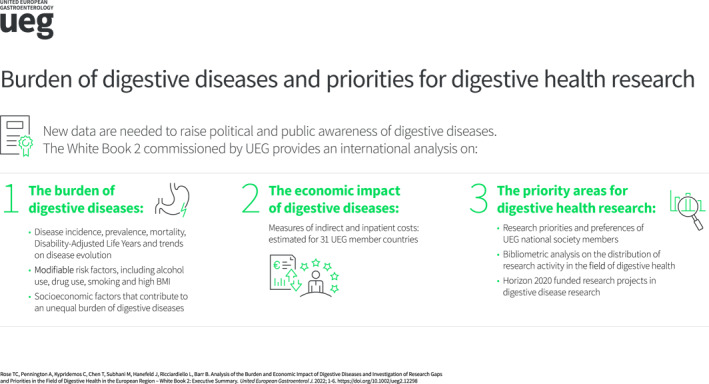

## INTRODUCTION

Despite their substantial burden, many digestive diseases are poorly understood, attracting relatively little attention in terms of policy, funding or research.^1^ United European Gastroenterology (UEG) commissioned the first White Book, published in 2014, which revealed important insights regarding the public health and economic burden of digestive disorders and health services across Europe.^2^, ^3^ In order to evaluate the current status, increase political and public awareness of digestive disorders and encourage digestive health research, UEG commissioned the White Book 2.

The White Book 2 updates analysis of the burden and determinants of digestive disorders, and explores unmet needs for digestive health research. It consists of two parts. Part 1 provides an international comparative analysis of the public health burden of digestive diseases and cancers, and analysis of the economic impact of digestive diseases amongst UEG national society member countries. In Part 2, research gaps and priorities in the field of digestive health are explored by consulting UEG national society members and examining distributions of research activity and European Commission funding for digestive disease related research.

It is intended that the findings from these reports will assist UEG in accelerating progress in reducing the burden of digestive disorders, and in identifying priority areas where research and investment are required. It is also hoped that the reports will be of interest to others, including national and specialist gastroenterology societies and policymakers. An overview of the objectives and key findings of the reports are summarised below. The full reports are available via the UEG website and can be accessed here: ueg.eu/white‐book2‐part1 and ueg.eu/white‐book2‐part2 (please see Appendix for details).

## PART 1—ANALYSIS OF THE BURDEN AND ECONOMIC IMPACT OF DIGESTIVE DISEASES IN THE EUROPEAN REGION

### Public health burden of digestive diseases and cancers

To explore the changing epidemiology of digestive disorders, an international comparative analysis examining variations in estimates of burden was performed for 44 UEG member countries, located within Europe and the Mediterranean area. Estimates produced by the Global Burden of Disease Study (GBD) 2019^4^ were utilised, alongside data obtained from published literature.

**FIGURE 1:**
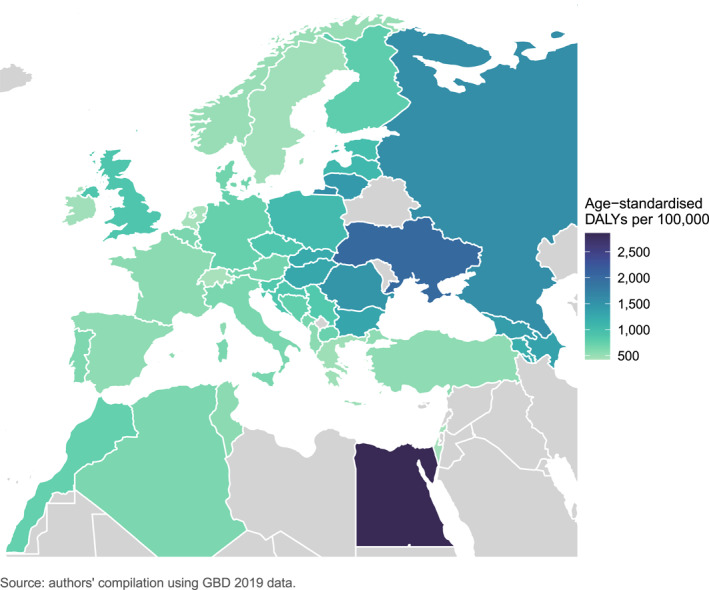
Age‐standardised Disability‐Adjusted Life Year rates for digestive diseases in 2019, for 44 UEG member countries

Key findings:

- Estimates from GBD 2019 suggest that digestive diseases are common and contribute to increasing levels of disease burden amongst UEG member countries, with the total number of incident cases, deaths and prevalent cases on the rise. The number of prevalent cases has increased by over a fifth since the year 2000, and currently over 300 million people are estimated to suffer from digestive diseases across UEG member countries.
- When accounting for population growth and ageing, for the member countries combined, age‐standardised incidence rates for digestive diseases overall have remained stable and age‐standardised mortality rates have decreased since the year 2000. Stable incidence and better survival have thus resulted in an increase in age‐standardised prevalence rates over time.
- For the member countries combined, age‐standardised incidence or prevalence rates have increased since the year 2000 for several digestive diseases, including chronic liver diseases, pancreatitis, gastroesophageal reflux disease, gastritis and duodenitis, paralytic ileus and intestinal obstruction, appendicitis, and vascular intestinal disorders.
- Previous studies have also found prevalence rates of eosinophilic oesophagitis and coeliac disease amongst children have increased over time. Incidence and mortality rates of diarrhoeal diseases among children aged <5 years have decreased since the year 2000 in several UEG member countries, likely explained by a combination of factors linked to socioeconomic development.
- The burden of digestive diseases as measured by Disability‐Adjusted Life Years (DALYs) tends to be higher amongst UEG member countries within Central and Eastern Europe compared to Western and Southern Europe, particularly for chronic liver diseases, pancreatitis, gastritis and duodenitis, appendicitis, inguinal, femoral, and abdominal hernia, vascular intestinal disorders, and peptic ulcer disease.
- Egypt has the highest age‐standardised DALY rate for digestive diseases of the member countries (Figure 1) which is to a large extent attributable to the high burden of liver disease due to viral hepatitis experienced within the country.
- Digestive cancers, together, are responsible for around a third of the total number of cancer related deaths amongst UEG member countries. Numbers of incident cases and deaths have increased since the year 2000 for all digestive cancers apart from stomach cancer. Estimated age‐standardised incidence and mortality rates have increased for liver and pancreatic cancers for most member countries since the year 2000, likely related in part to changes in the prevalence of modifiable risk factors.

### Economic impact assessment

To investigate the economic value of reducing the burden of digestive diseases, disease specific measures of indirect and inpatient health service delivery costs were estimated for 31 UEG member countries, mostly located within Europe, for which data were available.

Key findings:

- On average, the estimated cost of inpatient health service delivery (excluding treatment and diagnostics) for digestive diseases as a percentage of Gross Domestic Product (GDP) was 0.12% across the 31 countries.
- Based on past trends (using data up to 2019), inpatient health service delivery costs for digestive diseases are expected to decrease in many countries by the year 2035, possibly related to policies designed to improve efficiency in health service delivery in general. For some countries, however, inpatient health service delivery costs for digestive diseases are anticipated to increase.
- Estimated costs of lost output due to digestive disease related morbidity and premature mortality as a percentage of GDP were, on average, 0.22% and 0.25%, respectively across the 31 countries.
- To demonstrate the benefits of reducing digestive disease related premature mortality from a human capital perspective, if, across the 31 countries, digestive disease related premature mortality were reduced by 25%, 50% or 75% in 2019, the estimated savings from the prevention of productivity losses would have amounted to a total of €11.4, €22.8 or €34.2 billion, respectively. Reducing digestive disease related premature mortality by 25% in 2019 would have resulted in estimated savings of approximately €2.97 billion in Germany, €1.77 billion in the UK, and €1.4 billion in France, for example.

### Opportunities to reduce the burden

The overall lack of progress over the last 2 decades in reducing the incidence of digestive disorders underscores the need for greater adoption of effective preventative strategies amongst UEG member countries. To provide insights into evolving challenges and where the greatest health gains can be made, an examination of the proportion of digestive disorder burden attributable to modifiable risk factors was performed using GBD 2019 estimates, including an analysis of the relationship between human development and the burden of digestive disorders among European UEG member countries.

Key findings:

- Alcohol use contributes towards a large share of the overall burden of digestive disorders amongst UEG member countries. Whilst some progress has been made to reduce alcohol related burden, since the year 2000 several countries have experienced increases in the proportion of burden attributable to alcohol use for disorders such as liver disease, pancreatitis, liver cancer, oesophageal cancer, and colon and rectum cancer.
- Some relatively low alcohol drinking countries in parts of Northern Africa appear to have experienced increases in the proportion of digestive disorder burden attributable to alcohol use since the year 2000, which may have implications for public health policy planning within these countries.
- Almost all UEG member countries have experienced increases since the year 2000 in the proportion of burden attributable to high body mass index (BMI) for disorders such as gallbladder and biliary diseases, liver cancer, gallbladder and biliary tract cancer, pancreatic cancer, oesophageal cancer, and colon and rectum cancer.
- Since the year 2000, the majority of UEG member countries have experienced decreases in the proportion of burden attributable to smoking for disorders such as peptic ulcer disease, gallbladder and biliary disease, oesophageal cancer, liver cancer, stomach cancer, pancreatic cancer, and colon and rectum cancer.
- Lifestyle/behavioural risk factors for digestive disorders are socially patterned within most countries meaning that exposure to these risks and the harm caused by them is to a large extent determined by an individual's socioeconomic position. Interventions to address these risk factors which fail to consider the social, economic, environmental, cultural, and political factors which constrain people's choices and their opportunities to achieve healthy lifestyles, are likely to have limited success.
- Population‐level social and economic factors contribute to important differences in the burden of most digestive diseases and cancers between European countries and substantial health gains can potentially be achieved by considering policies which directly address the underlying social determinants of health. This may involve influential organisations such as UEG to encourage sectors beyond health to consider digestive health outcomes in policy and programme development.
- The coronavirus disease 2019 (COVID‐19) pandemic brought attention to the stark health inequalities experienced within countries and is likely to continue to present challenges for population‐health in the long‐term. Changes in alcohol consumption during the pandemic and emerging economic challenges such as rising inflation will likely exacerbate existing health inequalities, with implications for digestive health. As more data become available, an important avenue for future research will be to assess the impact of the COVID‐19 pandemic and its consequences on the burden of digestive disorders and socioeconomic inequalities in the burden within UEG member countries.

## PART 2—ANALYSIS OF RESEARCH GAPS AND PRIORITIES IN THE FIELD OF DIGESTIVE HEALTH IN THE EUROPEAN REGION

### Survey of the research priorities and preferences of UEG national society members

To gain the views of UEG national society members whilst creating an opportunity for national societies to communicate their research priorities to UEG and other societies, an online survey was conducted. The survey collected information on prioritised research topics and digestive disorders, and asked societies to rank the research areas previously identified as research priorities by UEG's specialist society members^5^ across three domains.

Key findings:

- In total, 33 responses to the survey were received from UEG national society members—a response rate of 73%. The 33 societies submitted 120 research priorities.
- The most prioritised research topics included drug therapy, diagnosis, and disease prevention research.
- The most prioritised disease categories included inflammatory bowel disease, digestive cancers, chronic liver diseases, diseases of the pancreas, and irritable bowel syndrome.
- Overall, inflammatory bowel disease drug therapy research was the most popular disease and topic combination identified for prioritisation by the societies, followed by digestive cancer prevention research.
- Of the research areas previously identified as research priorities by UEG's specialist society members, life‐style/nutrition/diet/obesity and primary prevention were, on average, the most highly ranked research areas in terms of relevancy to national health policy or national goals.
- EU trials/epidemiological studies/networks/surveys and primary prevention were, on average, the most highly ranked research areas in terms of the feasibility of conducting research in these areas.
- EU trials/epidemiological studies/networks/surveys and precision/personalised medicine were, on average, the most highly ranked research areas in terms of the potential to strengthen collaboration between partners from different organisations, disciplines or sectors.

### Distribution of research activity in the field of digestive health

To capture areas where research attention has been focused a bibliometric analysis was used to estimate quantities of published literature indexed within the MEDLINE database by topic and digestive disorder, and to identify areas that appeared under‐researched in relation to disease burden.

Key findings:

- A relatively large number of digestive disorder related academic journal publications were classified as pathology/physiopathology, diagnosis/diagnostic imaging, surgery, and drug therapy research.
- Amongst the digestive cancers, colon and rectum cancer and liver cancer had the greatest number of related publications, whilst oesophageal and gallbladder and biliary tract cancers had the fewest.
- Amongst the digestive diseases analysed, inflammatory bowel disease had the greatest number of related publications, and eosinophilic oesophagitis had the fewest.
- Inflammatory bowel disease appeared to be well researched in relation to burden compared to the other digestive diseases, whilst alcohol‐related liver disease appeared to be under‐researched in relation to the high level of burden associated with this disease.

### European Commission funding for digestive disease research within Horizon 2020

To identify and quantify European Commission funding for digestive disease related research, analysis of Horizon 2020 funded research projects was performed. Patterns of funding for digestive disease research in relation to disease burden in the European Union were examined and compared to funding granted for other diseases.

**FIGURE 2:**
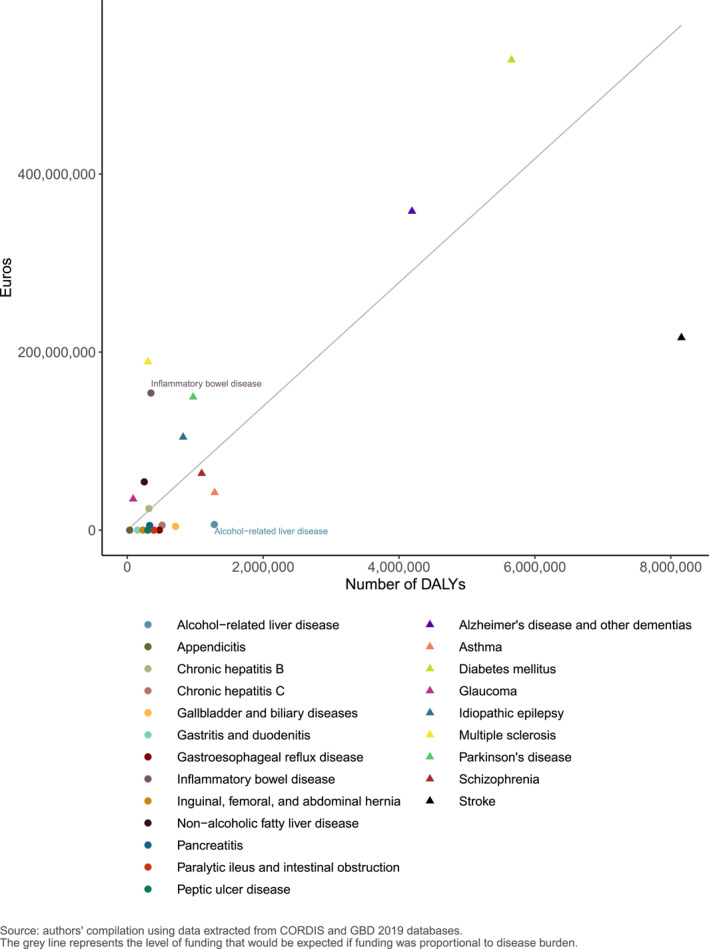
Relationship between European Commission funding for Horizon 2020 research projects and disease burden in the European Union (measured as Disability‐Adjusted Life Years)

Key findings:

- The digestive diseases that received the most research funding included inflammatory bowel disease, non‐alcoholic fatty liver disease, chronic hepatitis B and coeliac disease.
- Most of the digestive diseases analysed, however, received relatively small amounts of research funding and appeared to be under‐funded in relation to burden compared with other (non‐digestive) diseases (Figure 2).
- Gastroesophageal reflux disease, dyspepsia, peptic ulcer disease, and paralytic ileus and intestinal obstruction were amongst the digestive diseases that each received around €50,000 or less in research funding.
- Research investigating irritable bowel syndrome, one of the most common digestive diseases with limited available treatment options, was granted approximately €1.7 million in funding—equivalent to just over 1% of the amount awarded for inflammatory bowel disease research.
- Alcohol‐related liver disease research appeared to be under‐funded in relation to the high level of burden associated with this disease.
- If nine digestive diseases had received a proportionate amount of Horizon 2020 research funding relative to their disease burden, an estimated additional €283 million would have been allocated to these diseases in total, including almost €83 million for alcohol‐related liver disease research.
- Of the digestive cancers that were available to analyse, colon and rectum cancer and pancreatic cancer appeared to be under‐funded in relation to burden compared with some non‐digestive system related cancers.

### Recommendations for further research

- The analysis presented in the report is exploratory and intended to be used to initiate further discussions and more detailed investigations to further assist UEG in designing research agendas and advocacy strategies that are responsive to health needs and salient evidence gaps in the field of digestive health.
- Prioritised disease areas that appear under‐funded or under‐researched may represent research gaps that warrant greater consideration. Digestive diseases, such as irritable bowel syndrome, pancreatitis, and alcohol‐related liver disease, which received little in the way of Horizon 2020 research funding were highlighted as areas for prioritisation by the national societies. Additionally, disease prevention research appeared to be under‐researched yet regarded as an important area for prioritisation and may represent a promising avenue for development.
- Due consideration must be afforded to developing equitable research agendas and investment strategies given the low levels of research activity and funding for alcohol‐related liver disease—a condition which disproportionately burdens more socioeconomically disadvantaged groups, contributing to health inequities.
- Refinement of the potential areas for prioritisation that have been identified, into specific research questions for investigation can be achieved using systematic review methods and/or focused priority setting exercises.
- Gaining insight from patient groups who represent the intended beneficiaries of the research will be particularly informative. Capturing the views of marginalised and disadvantaged patient groups who may be more difficult to engage but likely have greater unmet health needs is especially important to inform decisions regarding avenues for further research.
- Additionally, further investigations are needed to identify barriers to conducting research in neglected areas, which will inform the development of effective strategies to encourage increased research activity and funding.
- Institutional body funding could be oriented not only at research grants but also for networking pan‐European activities with capacity‐building objectives which may provide long‐term benefits for digestive health research.
- Coordinated approaches to improve the surveillance of research activity and funding could also support research efforts by helping the research community to identify under‐researched areas and opportunities for collaboration.

## CONFLICTS OF INTEREST

The authors have no conflicts of interest to declare.

## Data Availability

The data that support the findings of this study are openly available. Details of the data sources are provided in the reports.

## Appendix

The full reports can be accessed below:

Rose TC, Pennington A, Kypridemos C, Chen T, Subhani M, Hanefeld J, Ricciardiello L, Barr B. *Analysis of the Burden and Economic Impact of Digestive Diseases in the European Region—White Book 2: Part 1*, 2022, ueg.eu/white‐book2‐part1.
Rose TC, Barr B. *Analysis of Research Gaps and Priorities in the Field of Digestive Health in the European Region—White Book 2: Part 2*, 2022, ueg.eu/white‐book2‐part2.
